# The implications of expectancy-value theory of motivation in language education

**DOI:** 10.3389/fpsyg.2022.992372

**Published:** 2022-11-08

**Authors:** Qi Wang, Mengchen Xue

**Affiliations:** School of International Education, Shandong University, Jinan, China

**Keywords:** expectancy-value theory, language education, motivation, cognitive processes, engagement and achievement

## Abstract

The successful performance of learners in any field of study, including a second/foreign language, is deemed as a pivotal concern in the educational system. Furthermore, the various learner variables, in particular, motivation should be taken into consideration, as a high level of motivation can yield many positive outcomes. Literature introduces the expectancy-value theory (EVT) as a recent approach to motivation, which has caught the attention of researchers. EVT as a basic and integrated paradigm helps the researchers and teachers to understand learners’ motivations and behaviors, and it has proved to be very helpful in understanding cognitive processes used by the learners, as well as their achievement. Based on this theory, successful performance, including the successful completion of the task and future aspirations, is mainly impacted by perceived expectancies regarding the outcome and value of tasks or domains. EVT can be viewed as a lens through which the aspects of motivation can be seen. This would pave the way for learners’ engagement and their achievement. EVT attaches great importance to learners’ reasonable expectations regarding the accomplishment of a goal. It also emphasizes figuring out the value of achieving the learning goal, which would enhance the motivation for L2 learning. The current review is aimed at examining how the expectancy-value motivational model impacts academic motivation, engagement, and participation in educational tasks, and learners’ academic performance.

## Introduction

Foreign language (FL) has proved to be very demanding, which entails unwavering perseverance, motivation, and efforts (Lou and Noels, 2020). In particular, motivation has been in the spotlight as one of the factors assumed to contribute to continued dedication to FL learning regardless of learners’ aptitude, L1, and the target language being learned (Dörnyei, 2008; Loh, 2019). As a mental process and an unobservable variable, motivation drives people to undertake goal-oriented actions and is deemed an essential contributor to the achievement of learning outcomes (Schunk, 2012; Alamer and Alrabai, 2022). Having no motivation has been mentioned as one of the main culprits regarding the poor performance of the learners in the context of L2. Studies show that academic success has emerged as a frequently cited phrase that is accompanied by multiple outcomes, such as scores, perseverance, learning, and goals (York et al., 2015; Goegan et al., 2020). Lacking motivation is one of the main culprits for the poor performance of learners in the L2 classroom (Daniels and Arapostathis, 2005). According to D’souza and Maheshwari (2015), learners must be driven by many sources of motivation when it comes to learning in L2 classes. Learners’ active engagement out of eagerness, curiosity, relishing, or the materialization of their own academic and personal goals are manifestations of intrinsic motivation (Deci and Moller, 2005; Abdullah et al., 2019). In all, learners with intrinsic motivation outperformed the learners with external motivation. They were more likely to seek long-term learning and show perseverance in education even after graduation. This was because they took part wholeheartedly in academic activities without being externally motivated (Brewster and Fager, 2000).

Educational psychologists have sought to focus on the possible factors contributing to some learners’ interest in learning more than others; moreover, the factors influencing their academic behaviors were of interest (Loh, 2019). Over the years, teachers and educators have been seeking to work out this problem, but it is still a serious challenge as before. This conundrum has been in the spotlight since the investigation of the relationship between language learning and motivation (Vanderbeen, 2005). Scholars have put forth multiple theories to account for what factors contribute to motivation and how people choose tasks. These theories have sought to untangle how people make efforts to obtain their objectives and how they control their sustained attempts to this effect (Yurt, 2015). Learners’ variations in terms of their persistence, eagerness for learning, motivation, and accomplishment in L2 learning might be accounted for by their perceptions of their own ability, expected achievement, and subjective task values (Wigfield and Eccles, 2000) that are concerned with expectancy-value theory (EVT), which is an approach to the investigation of academic motivation. This is claimed to justify learners’ task selection, sustained learning, and academic performance (Wigfield and Eccles, 2000; Wigfield and Cambria, 2010). The expectancy-value theory makes a connection between achievement, perseverance, and learners’ expectancy-related beliefs and their perceptions regarding the task value (Eccles, 2011). Focusing on learners’ psychological thinking (Eccles, 2009), EVT seeks to uncover how learners’ perceptions are formed over time by different personal and social factors (Eccles and Wigfield, 2002). Sequentially, these insights, impact academic choices and achievement results. Moreover, EVT makes a distinction between learners’ expected success (expectancy) and the extent to which they place value over the topic (value) as the main components of motivation. Indeed, EVT has been developed as a hypothetical frame aimed at painting a comprehensive picture of motivation, its underlying factors, and learners’ sustained undertaking.

Moreover, EVT (Chen and Sheu, 2005; Mori and Gobel, 2006; Loh, 2019) makes a distinction between learners’ expectations of success, expectancy, and how much value they place on the subject, value, as core aspects of motivation (Lee and Bong, 2019; Loh, 2019; McEown and Oga-Baldwin, 2019). The first one is expectancy, which refers to learners’ perceptions regarding their future success in doing a task. This is also known as their expectancies for success (Eccles and Wigfield, 2020). Value beliefs refer to the degree of importance and value placed by the learners on a task (Eccles et al., 1983; Meece et al., 1990). Put it another way, an individual who thinks that his/her engagement in a task would lead to a positive outcome, but he/she lacks a strong reason to do so will avoid making efforts. Also, if he/she deems an important task to be unattainable, the individual tends to take part in an additional task with a greater expectancy of success (Putwain et al., 2019).

The expectancy-value theory is a useful framework through which one can understand how learners perceive themselves and their abilities, as well as how others see them. Moreover, research has proved that educational context influences learners’ academic choices, goals, and success (Rosenzweig et al., 2019). Notwithstanding some studies conducted on the EVT of motivation in some areas (e.g., mathematics, ESL, and technical fields) (Jones et al., 2010; Perez et al., 2019), few studies have investigated the theory in the area of FL (Tremblay and Gardner, 1995; Nagle, 2021). The majority of EFL students may find EL courses very valuable. Learners’ perception of L2 learning as an important endeavor inspires them to invest both time and energy to develop their competence. In China, English enjoys a special status for both government and people and it has a key function in educational success, trade, public administration, and technology. English is considered both a school subject and a yardstick for sophisticated education. More importantly, as an international language, English connects China with the other section of the world (Tan et al., 2017). It is anticipated that all learners study and use English well as English enjoys a distinctive value in teaching.

The expectancy-value theory is very promising for opening a new window unto motivation and L2 learning as it formulates testable hypotheses about the various aspects of motivation that can be the predictor of achievement. Moreover, this framework aimed to deal with learners’ motivation, perseverance, and achievement. However, no review has dealt with the investigation of EVT in the L2 learning context.

## Review of the literature: An introduction to expectancy value theory

Historically, the expectancy and value construct date back to the time when achievement motivation emerged as a research interest many years ago (Higgins, 2007; Wigfield et al., 2016). Building on the research findings of the earlier works conducted by Lewin and Tolman, Atkinson (1957) developed an expectancy-value model to capture people’s different achievement-related behaviors, including perseverance, sustained efforts for accomplishment, and selection of achievement tasks. These behaviors were influenced by three main components related to a person: motives, perceived likelihood/expectancy of achievement, and the incentive value attributed to an activity. These components, namely, expectancy and value emanate from people’s image of previous situations and socialization processes (Wigfield and Eccles, 1992), which result in the formation of task-specific convictions, such as capability beliefs, the perceived complexity of various activities, people’s goals, and affective memories that, in turn, impact the development of expectancy and value beliefs (Eccles et al., 1998).

Based on this theory, a learner’s motivation is driven by two subjective beliefs (Eccles and Wigfield, 2002) and in line with EVT, there is a close connection between achievement and its corresponding behavior and expectancy and value perceptions (Trautwein et al., 2012). Expectancies are concerned with an individual’s prediction for achievement or beliefs about how well an individual will deal with a prospective task. As pointed out by Eccles and Wigfield (2002), expectancy for success serves as a contributor to driving performance, attempts, and perseverance regarding tasks. This type of expectancy is often assessed through questionnaires of self-efficacy or perceived ability, both of which cannot be empirically distinguished by the learners (Wigfield et al., 2006). Both competency and efficacy beliefs are essential parts of the expectancy model (Wigfield et al., 2004). According to Eccles et al. (1983), expectancies consist of one’s perceived ability, perceived difficulty of a task, a mentality of expectations raised by others, attribution of causes, and locus of control. Indeed, ability and efficacy principles are sources in the expectancy model (Eccles and Wigfield, 2002). Ability self-concept was described as learners’ mental judgment of the degree of their ability to accomplish the task, whereas perceived difficulty of tasks was characterized as learners’ mental judgment of the difficulty involved in the successful completion of the task (Flake et al., 2015; Rosenzweig et al., 2019). Leaners who enjoy a positive perception of their ability are convinced that they can be successful in learning a language (e.g., because they had previous successful experience regarding language learning), whereas learners who perceive the task to be highly difficult deem their course to be challenging and tricky. Self-concept of ability in combination with the perceived difficulty of the task is at play to answer the question “Can I do this task?”(Schunk et al., 2007).

Another group of learners’ perceptions has to do with the extent to which they value particular tasks or subjects. Based on a model developed by Eccles and Wigfield (2002), there are two types of expectancies, namely, beliefs regarding one’s abilities and expectancies for success. The former is concerned with an individual’s present perception of competence to carry out a task. Expectancies for success have to do with reflecting on how successful a person thinks his/her future will be. Generally, subjective task values are concerned with the “values” placed by a learner on a particular task, which may impact his/her persistence or choice of a particular task or activity (Wigfield et al., 2016). The term “subjective” invokes a learner’s personal perceptions regarding an activity; therefore, values mean different things to different learners. Subjective task values involve the various qualities of particular activities which may impact the degree of willingness or motivation to carry out the activities (Wigfield and Cambria, 2010). Prior research shows that subjective task values can predict motivation better than expectancy and self-efficacy theories (Xiang et al., 2003). Expectancies are concerned with broad areas, which, in turn, are associated with general consequences while self-efficacy concerns more specific activities that are related to one’s ability to obtain a specific result. For instance, an expectancy measure can assess a person’s abilities in specific areas (English), and this measure can be used to make a prediction regarding the scores on that subject. A self-efficacy indicator can assess a person’s abilities to carry out a precise activity in a class, and the reactions can be employed to predict the performance on this specific task (Hulleman et al., 2016).

According to EVT, four categories of subjective task values can be identified (Eccles and Wigfield, 2002). One of them is intrinsic value, which is also known as interest value. It is concerned with the natural relishing one can gain from doing the task and corresponds with intrinsic motivation in self-determination theory (SDT) (Anderman, 2020). The ideas of intrinsic and extrinsic motivation in SDT were taken from the idea that diverse kinds of achievement motivation can be defined by fluctuating levels of self-determination articulated as objective and peripheral (Alamer and Lee, 2019). This theory attributes intrinsic motivation to the endeavors and tasks carried out for their natural value (Ryan and Deci, 2020). Accordingly, the students driven by intrinsic motivation try to do the task as they are interested in doing it. Another concept is utility value which refers to the usefulness students find in completing a task, which serves their short- or long-term objectives (Eccles and Wigfield, 2020). Put it another way, the utility value has to do with the people’s perceptions of a task’s relevance or usefulness for their future goals. Learners who enjoy high utility value find English important as they should necessarily pass the present English course to reach the next grade. These components are combined to answer the question, “Do I want to do this task, and why?” (Schunk et al., 2007). The more relations between the course material and the learners’ everyday lives, the greater the utility value and; as a result, the better the learning achievement (Hulleman et al., 2017).

The other is the attainment value which is concerned with the significance of the successful completion of a task, which has to do with the learner’s personal goals, such as gaining proficiency and skills (Eccles and Wigfield, 2002; Arens et al., 2019) that refers to the learners’ efforts made to complete a task perceived by them to promote their identity (Perez et al., 2019). Attainment value was considered as the perceived personal importance and recently it has been found to reflect identity-based importance (Eccles and Wigfield, 2020). Tasks play an important role given that people see them as having a pivotal role in enabling them to demonstrate or confirm significant aspects of themselves (Wigfield et al., 2016). Moreover, as a motivation-related notion, the cost is generally viewed as the time and energy people invest in carrying out an activity (Eccles, 2009; Flake et al., 2015). Cost involves more than the financial burden incurred through spending time and energy on an activity. Cost refers to the external burden that may emanate from doing an activity (e.g., the cost of time). Cost is the least researched factor of sub-concept (Wigfield et al., 2009; Vernadakis et al., 2014; Wang and Guan, 2020) that has a noteworthy function in the value system, which is concerned with the extent to which one makes efforts, especially in the face of adversaries (e.g., stress, fear, apprehension of both failure and success), and missing out on other opportunities due to the selection of a particular task (Eccles and Wigfield, 2002; Plante et al., 2013). While making decisions, learners are also gauging the degree of their motive and sustained efforts they would invest in accomplishing the particular learning task, which is viewed by learners as an element regardless of expectancy and value (Flake et al., 2015).

In accordance with EVT, several factors determine learner motivation, including expectancy beliefs, the value one places on a task, and how the task is perceived. As pointed out by Eccles and Wigfield (2002), these factors have a direct impact on learner achievements and behavior choices. This theory takes account of various learners’ factors, achievements, expectancy beliefs, perceived values, achievements, and experiences; consequently, it serves as a useful framework to account for these important issues. Based on this model, learners’ motivation can be a predictor of their learning and their behaviors out of school, and that learning over time is a predictor of their achievement-related behavior (Eccles and Wigfield, 2002). Moreover, it can be stated that the interactive teacher-student relationships and also teacher stroking behavior enable not only learners but also the teacher to have better communication leading to a more attractive instructive development (Pishghadam et al., 2019, 2021).

## Conclusion

As sophisticated knowledge and the acquisition of skills entail sustained effort and perseverance, educators should pave the way for a positively motivating experience for the learner (Alexander, 2006). In the majority of situations, motivation plays a determining role in the quality of learning; however, figuring out the connections between numerous parts of motivation and L2 learning outcomes is the first step in effectively enhancing motivation. Indeed, in the context of classrooms, learner motivation drives them to carry out their learning tasks and activities on the daily basis. Drawing on EVT, this review dealt with the various aspects of motivation that could serve as predictors of success, perseverance, and achievement when it comes to L2 learning. Indeed, language achievement has been considered to be meaningfully increasing among the learners, and such development was related to the development in motivation, so indicating the idea of an “immediate resource” that each aspect hinges on at a particular time point (Alamer and Alrabai, 2022). Based on these insights, some recommendations were made to help learners develop and sustain their L2 learning motivation (Nagle, 2021). The inclusive comprehension of the potential contributors to learners’ motivations and intentions and their persistence in academia requires the consideration of learners’ perceptions and values.

According to the EVT model, expectancies for success and subjective task values directly influence persistence, selection, and the level of motivation (Metallidou and Vlachou, 2007). Expectancies for success, which are the first component of the EVT model, have to do with the beliefs held by an individual regarding one’s ability to carry out a task at present or in the future. It also concerns the perceived effort of the task (Matusovich et al., 2008; Wigfield and Cambria, 2010). One can use the term expectancy to mean both expectancies of one’s successful performance and ability beliefs. As learners’ expectancy beliefs are concerned with learners’ present capabilities and their successes in the future, as well as the relationship between abilities and motivation, one can assume that learners’ motivation may undergo some changes over time. The factors which can make these changes include the impact of test scores and competitions. L2 achievement can enhance positive educational motivation since learners tend to expend more time and energy when they have mastery over a subject. So, it should be tried to arouse emotions in language circumstances that refers to the notion of emotionacy as it might upsurge learners’ confidence and compassion that might increase their motivational energy and assist language learning process (Pishghadam et al., 2016). Moreover, as research findings in the field of educational psychology show, expectancy of success can serve as an important predictor of academic achievement. It consequently provides a significant influence to the psychology of language learning (Mercer et al., 2012). Research also shows that value appraisals can predict motivated behaviors and persistence (Guo et al., 2017), as well as the relationship between curriculum design and learners’ lives. The great level of utility value leads to a great level of learning.

For instance, learners may think they are proficient in some skills. This, in turn, may make them believe that their performance in the related activities would be successful, as well. Such expectancy is likely to push the learner to choose specific classes in university, followed by being admitted into that field. Therefore, there are some features shared by both expectancies and other psychological constructs already studied (e.g., self-efficacy that is concerned with the learner’s perceptions of his/her successful completion of a task (Bandura, 2010). In accordance with EVT, learners’ self-efficacy, i.e., their perceptions of their current abilities, are concerned with their later expectancies regarding success and task value (Wigfield and Eccles, 2000). Task value is related to learner interest and positive emotions in class, which has also been called intrinsic value (Fredricks et al., 2004). There is some overlapping between intrinsic value and emotional engagement. The latter involves interest and enjoyment. As a result, based on this theory, self-efficacy results in emotional engagement. Furthermore, self-efficacy and emotional engagement result in behavioral engagement, which involves sustained effort, focused attention, compliance, etc. These behaviors are some instances of achievement-related choices, which are indicative of the learner’s desire to succeed and fulfill classroom assignments. Therefore, EVT assumes that learner behavioral investment appears as a consequence of an internal motivation to learn. Such a type of motivation is characterized by self-efficacy and emotional engagement that they result in learners’ achievement. Also, engagement with its sensory nature, relies on the psycho-linguistic notion of emotioncy, which is assumed to utilize great impact on motivation that also affect learners’ success (Miri and Pishghadam, 2021).

Attainment value is another dimension, which has to do with learners’ self-schemas. In other words, given that learners’ choices and performances may reveal some angles of their identity (e.g., masculinity or competence), they are likely to perform a certain behavior driven by certain motivation. Utility value is likely to impact learner’ motivation as if L2 learners see language learning as an important contributor to their success in the future, they take these subjects and study them (Black et al., 2010). Utility value is concerned with the learner’s perceptions of the usefulness one obtains due to the performance of a task. Given the overwhelming power of extrinsic performance rewards, one can equalize utility value with extrinsic motivation, which is one of the components of SDT (Trautwein et al., 2012). According to Ryan and Deci (2020), intrinsic value corresponds to intrinsic motivation, while utility values correspond to extrinsic motivations. This is because both values have to do with relishing in the pursuit of the subject and future goals, respectively.

The new version of EVT emphasizes learners’ motivational beliefs and maintains that such beliefs impact learners’ achievement-related behaviors, academic goals, academic options, participation, and successful performance (Trautwein et al., 2012; Guo et al., 2017; Putwain et al., 2019; Zeynali et al., 2019; Wang and Guan, 2020). Research evidence shows that expectancy of success and value can drive a multitude of educational outcomes. In a nutshell, it can be argued that the two components of this model, namely, expectancy for success and task values, mutually reinforce each other. Learners tend to take part in the tasks they value. Also, their participation in those activities will impact their expectancy for success. This, in turn, leads to some changes in values (Nagle, 2021). Learners make conscious or unconscious decisions on how they would invest their energy and time, that is, their level of engagement. Individually, these decisions are mainly impacted, at least in the early stages, by the degree of confidence a learner has about successful performance on a task, the valuable chances the task provides for the person in terms of the amount of input and control, their enthusiasm for the task and to what extent the learners feel prepared to address the task. For example, people may choose to acquire an L2 since they find it appealing or useful or merely due to a requirement. Their good performance on L2 courses will lead to an increase in their expectancy of success. This, in turn, would yield more enjoyment of L2 tasks (intrinsic value). By the passage of time, enjoyment may induce the impression that it is personally important to become a competent L2 speaker (attainment value), particularly, if L2 learning is seen as an inseparable part of becoming an effective learner and/or a global citizen.

Literature shows that expectancy and attainment values can mutually enhance each other in L2 learning situations. Put it another way, learners’ high expectations of successful performance can neutralize the negative impacts of low attainment value on foreign language success. As a result, teachers are recommended to emphasize improving attainment value as their behavior is an effective way to enhance the foreign language achievement of learners (Pishghadam et al., 2021). Meanwhile, attempts made to enhance learners’ expectancies of success can be seen as an effective strategy to boost learners’ low level of attainment value, which, in turn, enhances their foreign language achievement. The expectancy component is concerned with learners’ beliefs regarding their own competence and self-efficacy (Wigfield and Eccles, 2000; Eccles and Wigfield, 2002), while, the value component has to do with the rationale one has for participating in a specific task.

Following the EVT, cultural environment and social beliefs and behaviors impact learners’ perceptions of their experiences (attribution), their perceptions of abilities, and their purposes. These variables, in turn, impact their predictions of success and the value they place on the tasks. This results in the emergence of achievement-related choices and performances. Put it another way, a meta-analysis of the previous studies conducted on motivation shows that learners are seen both as decision-makers and meaning creators; furthermore, the learner variables such as expectations, perceptions, and social backgrounds play an important role in their achievement motivation (Cho and Hwang, 2019).

## Pedagogical implications and suggestions for further research

It is assumed that based on EVT theory, learners always engage in constant assessment of their competence with respect to the task at hand to learn successfully (Eccles et al., 1984). Meanwhile, learners’ expectations of the value involved in learning tasks; the values placed by learners on tasks function as contributors to meaningful engagement in learning (Wigfield, 1994). They offer an essential source of motivation for learners to get past difficulties, which will yield positive outcomes related to the learning experience. Learners’ expectancy and value beliefs can effectively predict academic outcomes and success. Moreover, being proficient and having the skills to learn and persevere amid various challenges are pivotal to learners’ future success (obtaining educational credentials, picking a job, or advancing in long-term employment). Therefore, the adjustment of the associations between expectancy-value motivation and learning consequences requires the identification of the origins of expectancy and value that can be changed. It should be noted that motivation is considered a changeable, collaborative construct (Dörnyei et al., 2015; Fan and Wang, 2022), so curriculum designers, teachers, policy-makers, and researchers can bridge the achievement gaps, driving more learners to make sustained efforts in academic situations, both in the short and long term.

Task values mutually impact each other and they also have interactions with the expectancy of successful performance over time, which drives motivation and learning. Therefore, studies in the future need to investigate EVT constructs longitudinally for purpose of understanding how changes in task values and expectancy influence efforts and achievement. The present investigation reviews the previous studies; however, more investigations, particularly, qualitative and mixed-methods ones must be conducted to shed light on motivation–persistence–achievement links. Prospective investigations can examine the potential interplay among the value constructs, along with other motivational variables as mediators. These studies contribute valuable info to the literature. Moreover, experimental research needs to be carried out in this regard as well. This type of research makes changes to motivational factors as precursors of academic procrastination and persistence in EVT. Also, taking into account the variables such as cultural milieu and gender socialization can provide some insights into the efficacy of motivation in L2 learning.

## Author contributions

Both authors wrote the manuscript and approved its final submission to Frontiers in Psychology.
